# P03.05. Creating an interprofessional curriculum in integrative medicine for medical, nursing, pharmacy, and dentistry students: curriculum mapping strategies

**DOI:** 10.1186/1472-6882-12-S1-P258

**Published:** 2012-06-12

**Authors:** S Adler, Y Coulter

**Affiliations:** 1 University of California, San Francisco, San Francisco, USA

## Purpose

An increasing number of academic health institutions are committed to integrative medicine (IM) principles, including partnership between patient and practitioner; collaborative, interprofessional health care; and promotion of health and the prevention of illness. The objective of this five-year project is to develop, implement, evaluate, and disseminate a multidisciplinary, interprofessional curriculum in IM. Our first aim was to map the existing IM curricula to examine the content longitudinally within each school, as well as across schools. To do this, we added to traditional curriculum mapping methods by eliciting faculty and students’ perspectives through interviews.

## Methods

We formed multiprofessional working groups of IM educators, practitioners, and students. Our enhanced curriculum mapping process included collection of online and paper-based syllabi and other materials from courses, as well as semi-structured interviews with course faculty, members of an IM student interest group, and other health professions students. We assembled a database of the collected components to examine content differences and areas of overlap within and across the schools. Three investigators coded transcripts independently, identified themes, and reconciled differences.

## Results

The curriculum map revealed isolated and scattered content within each school and different educational emphases across the health professional schools. Faculty and learners perceived the absence of a systematic and coordinated approach to IM curricula and emphasized the lack of both sequential development and an iterative pattern within and across schools.

## Conclusion

Traditional curriculum mapping makes curriculum development more effective and efficient. Adding faculty and student interviews helped contextualize the mapped results. An unanticipated benefit of our enhanced approach to curriculum mapping was that it generated enthusiasm and fostered collaboration for the interprofessional curricular innovation. Using IM principles to educate and engage interprofessional learners enables individuals to work together more effectively; share problem-solving and decision-making tasks; and integrate disparate knowledge structures into a single action plan.

